# Paneth cell ontogeny in term and preterm ovine models

**DOI:** 10.3389/fvets.2024.1275293

**Published:** 2024-01-22

**Authors:** Geoanna M. Bautista, Anjali J. Cera, Rebecca J. Schoenauer, Michele Persiani, Satyan Lakshminrusimha, Praveen Chandrasekharan, Sylvia F Gugino, Mark A. Underwood, Steven J. McElroy

**Affiliations:** ^1^Department of Pediatrics, University of California, Davis, Sacramento, CA, United States; ^2^Stead Family Department of Pediatrics, University of Iowa, Iowa City, IA, United States; ^3^Department of Pediatrics, University of Buffalo, Buffalo, NY, United States

**Keywords:** Paneth cell, immature intestine, perinatal bradycardic stress, preterm, ovine

## Abstract

**Introduction:**

Paneth cells are critically important to intestinal health, including protecting intestinal stem cells, shaping the intestinal microbiome, and regulating host immunity. Understanding Paneth cell biology in the immature intestine is often modeled in rodents with little information in larger mammals such as sheep. Previous studies have only established the distribution pattern of Paneth cells in healthy adult sheep. Our study aimed to examine the ontogeny, quantification, and localization of Paneth cells in fetal and newborn lambs at different gestational ages and with perinatal transient asphyxia. We hypothesized that ovine Paneth cell distribution at birth resembles the pattern seen in humans (highest concentrations in the ileum) and that ovine Paneth cell density is gestation-dependent.

**Methods:**

Intestinal samples were obtained from 126–127 (preterm, with and without perinatal transient asphyxia) and 140–141 (term) days gestation sheep. Samples were quantified per crypt in at least 100 crypts per animal and confirmed as Paneth cells through in immunohistochemistry.

**Results:**

Paneth cells had significantly higher density in the ileum compared to the jejunum and were absent in the colon.

**Discussion:**

Exposure to perinatal transient asphyxia acutely decreased Paneth cell numbers. These novel data support the possibility of utilizing ovine models for understanding Paneth cell biology in the fetus and neonate.

## 1 Introduction

The intestinal tract is the largest surface of the human body that is exposed to the external environment. The role of the intestine is unique as it must facilitate nutrient absorption while simultaneously protecting the host from the multitude of antigens and microbes present in the intestinal lumen. Specifically, the preterm infant has an underdeveloped intestine with a dysregulated immune response and dysbiotic pro-inflammatory microbiome. This leads to increased intestinal inflammation and permeability in the gut. These derangements predispose preterm infants to bacterial translocation and invasion of the intestinal epithelium which can lead to conditions such as neonatal necrotizing enterocolitis (NEC) (1, 2). NEC is a devastating disease primarily affecting premature neonates and is characterized by massive intestinal inflammation, tissue necrosis, and muli-system organ failure.

The Paneth cell is a compelling example of this abnormal host-microbial interaction (3–5). Paneth cells were first described by Gustav Schwalbe and Josef Paneth in the 19^th^ century (6, 7) and are located at the base of the intestinal crypts of Lieberkühn (8, 9). Paneth cells contribute to the intestinal immune response by releasing their granules which contain antimicrobial peptides (defensins and cathelicidins) and immune modulators (cytokines, immunoglobulins, lysozyme, and bacteriolytic enzymes) that help to regulate both the bacterial composition of the intestine and homeostasis of the adjacent intestinal stem cells (6, 10, 11).

Paneth cells play a critical role in the development of NEC (3, 12–14). Our group and others found that human infants diagnosed with NEC have fewer Paneth cells than age-matched controls (13, 15–18), and experimental NEC can be induced in neonatal mice following Paneth cell disruption in the presence of dysbiosis (12, 14, 19, 20). In addition, the propensity of preterm infants to develop NEC may be partly explained by Paneth cell biology, as Paneth cell numbers and function develop during intestinal maturation, and competence does not develop until close to full term in humans and several weeks after birth in mice (8, 21, 22). However, the study of Paneth cell development in the preterm infant is technically challenging due to a scarcity of tissue samples and the limited availability of relevant reagents. Thus, animal models are frequently used to study how Paneth cells impact the immature intestine and the development of diseases such as NEC (23–25). Most often, mouse and rat models are utilized due to their low cost, easy maintenance, fast reproductive rates, large litter sizes, and the ability for genetic manipulation. However, rodent models are limited for studies of the immature intestine. Newborn mice have a relatively immature intestinal tract compared to humans, lacking intestinal crypts and Paneth cells at birth and developing their intestines postnatally (8, 21). To adjust for this developmental difference, many rodent models of NEC need to be performed 1–2 weeks after birth (14, 19, 20, 25–28). In addition, rodent models can pose size-related challenges that make certain types of imaging, vascular cannulation, and sequential blood sampling difficult. Thus, using large animals, such as sheep, is an attractive potential adjunct to rodent studies.

In humans, Paneth cell populations first appear in the first trimester around 13.5 weeks of gestation, begin to function around 22–24 weeks, and continue to increase in density and maturity throughout gestation (8, 21, 22). Humans have the highest density of Paneth cells in the distal portion of the small intestine (8), and Paneth cell metaplasia is sometimes noted in the colon (29). Mice, however, do not develop Paneth cells until 7–10 days after birth, depending on the strain of mice (30, 31). In addition, while mice have the highest density of Paneth cells in their distal small intestine, they can also be found proximally (32). Further, some mammals do not express Paneth cells at all (6, 33). Thus, to study Paneth cell biology in the immature intestine, it is crucial to understand the ontological timing of Paneth cell development and the distribution and density of Paneth cells throughout the intestine.

While ovine models are not ideal for understanding nutrient absorption or microbiome studies due to their rumen, which performs a large portion of metabolism and harbors a significantly different microbial composition compared to humans (34), they have been successfully utilized to understand inflammatory diseases of the fetus and newborn (35, 36). In addition, ovine models played an important role in understanding fetal physiology and response to asphyxia (37). However, understanding of Paneth cell development in ovine models, specifically in term and preterm lambs, is limited. Previous studies have been limited to healthy adult sheep (38) or only assessed the mRNA expression of lysozyme in a limited number of lambs (39). Additionally, no current research has demonstrated the effect of bradycardia on Paneth cell density throughout the lamb small intestine. Our study aimed to examine such issues, and we hypothesized that ovine Paneth cell distribution patterns at birth closely resemble the distribution pattern seen in humans and mice, with the highest concentrations in the ileum and are gestation dependent. Lastly, we hypothesized that Paneth cell concentrations are depleted when the lambs are exposed to birth stress conditions.

## 2 Materials and methods

*Animals*: All animal experiments were approved by The Institutional Animal Care and Use Committee (IACUC) at the State University of New York at Buffalo (Protocol # PED 10085 N). All experiments were performed following relevant guidelines and regulations in accordance with ARRIVE guidelines. Time-dated Mixed Dorset pregnant ewes were obtained from May Family Enterprises, Buffalo Mills, Pennsylvania, USA.

*Sheep experimental conditions:* Fetal lambs were delivered via cesarean section following maternal induction with ketamine (4 mg/kg) and diazepam (0.5 mg/kg) and mechanical ventilation as previously described (40–42). Newborn sheep delivered between 123–126 days of gestation were classified as preterm (n = 15) and sheep delivered between 141–147 days of gestation were classified as term (n = 8). The delivered lambs were intubated, lung liquid was drained by gravity, and ventilated with conventional ventilation for 2 h. All preterm lambs received surfactant (3 cc/kg, calfactant – ONY Inc., Amherst, NY) before initiation of ventilation. In all lambs, intestinal tissue samples were obtained during euthanasia using intravenous pentobarbital (100 mg/kg) (Fatal-Plus, Vortech Pharmaceuticals, Dearborn, Michigan, USA).

*Induction of perinatal bradycardia:* In 6 preterm lambs, bradycardia was induced via cord occlusion prior to delivery until a steady heart rate (HR) of 60 bpm was achieved. Lambs were then ventilated, given surfactant, and resuscitated following standard neonatal resuscitation procedures until spontaneous HR > 100 bpm was achieved and systolic blood pressures were > 40 mmHg for 2 h.

**Figure 1:**
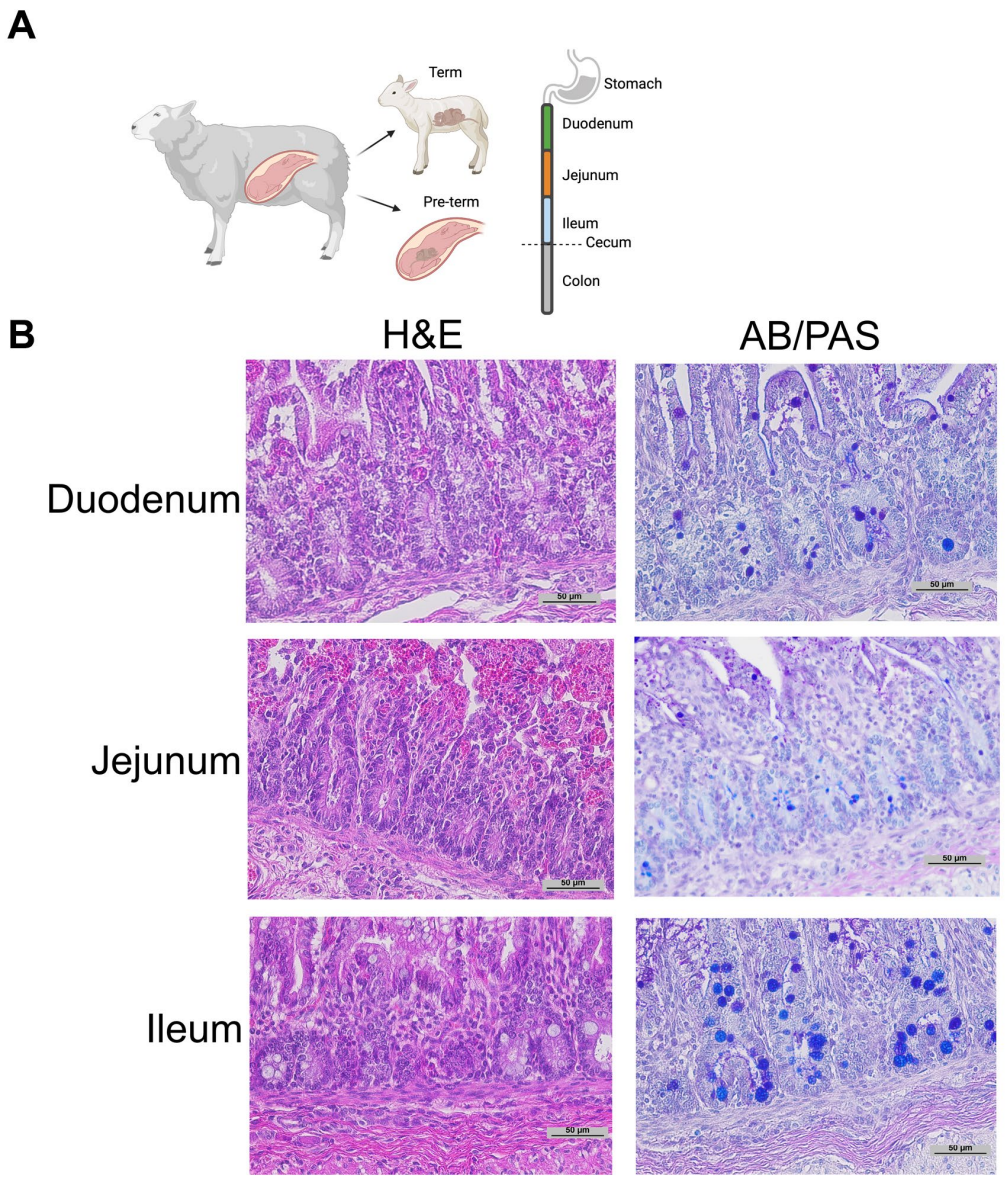
Representative histology. **(A)** Graphical diagram of methodology. **(B)** Representative term intestine stained with H&E and Alcian Blue Periodic Acid Schiff stain at 20 x magnification.

**Figure 2:**
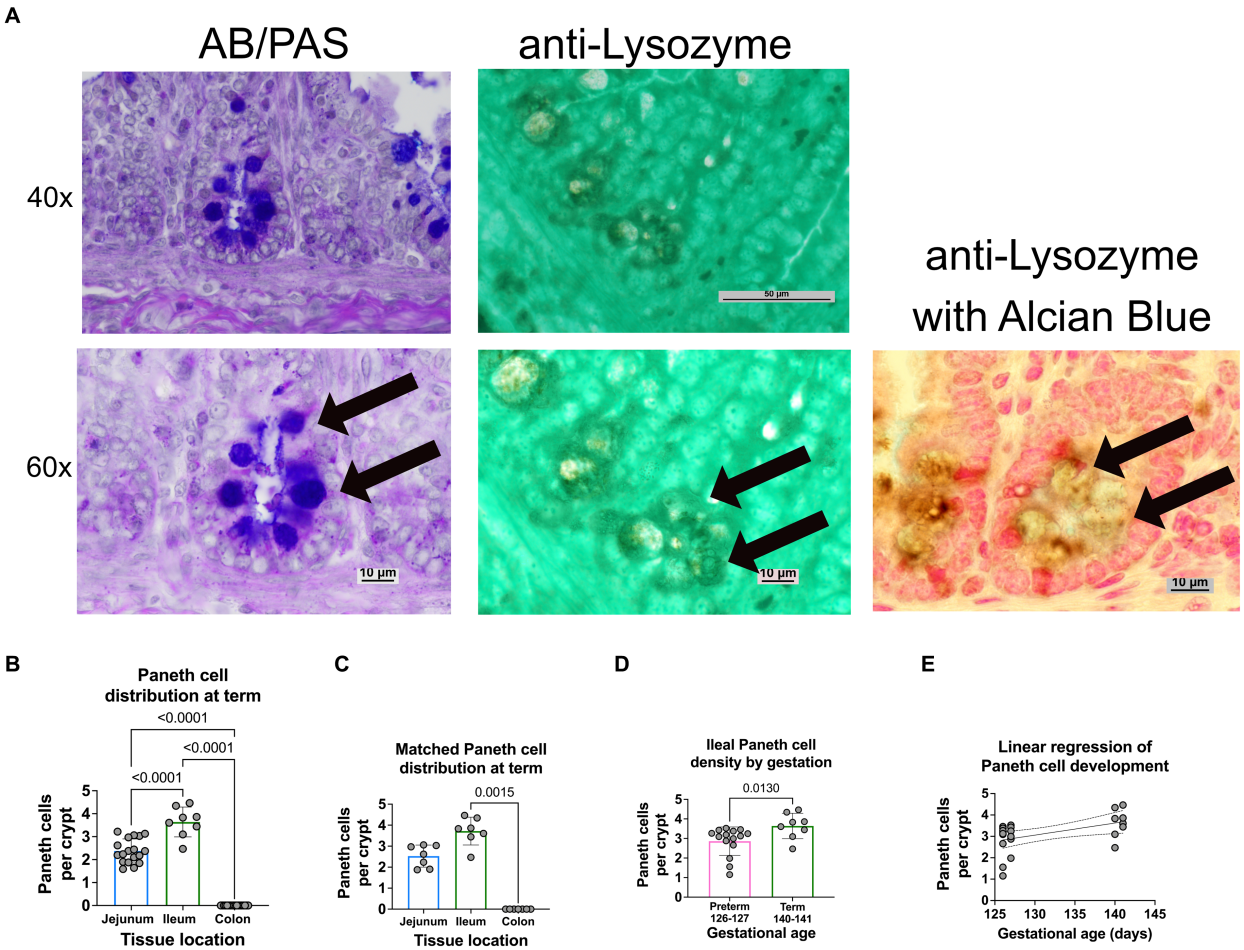
Paneth cell density varies by region and gestation along the small intestine of a newborn sheep. **(A)** Representative histology of preterm ileal samples at 40x and 60x stained with AB-PAS and anti-lysozyme techniques. Paneth cells are designated by arrows at 60X. **(B)** The ileal portion of the small intestine contained the highest average density of Paneth cells (3.72 Paneth cells/crypt, *n* = 8), followed by the jejunal small intestine (2.3 Paneth cells/crypt, n = 18). The colonic samples had no visible Paneth cells (0 Paneth cells/crypt, *n* = 18) (*p* < 0.0001 for all comparisons). **(C)** Matched samples that contained all three intestinal segments (*n* = 7) showing a similar distribution to the entire study population. In the matched series, the ileal Paneth cell numbers significantly differed from the colonic samples (*p* = 0.0015). There was a nonsignificant trend for increased Paneth cells in the ileum compared to the jejunum (*p* = 0.5). **(D)** Full-term control samples (140–141 days of gestation, *n* = 8 from Figure 1) had an average density of 3.72 Paneth cells/Crypt as compared to preterm ileal samples (126–127 days of gestation, *n* = 15), which had an average density of 2.86 Paneth cells/crypt (*p* = 0.013). **(E)** A linear regression model was calculated to interrogate the correlation between gestational age and Paneth cell density. As shown, there is a direct correlation between increasing gestational age and Paneth cell density (*p* = 0.016, R^2^ = 0.25, Sy.x = 0.7).

*Histology*: Intestinal tissue was collected and fixed in neutral buffered 10% formalin, embedded in paraffin, and sectioned at 5 mm thickness. Intestinal segments were defined as proximal 1/3 of the small intestine—duodenum; mid 1/3 of the small intestine—jejunum; distal 1/3 of the small intestine—ileum; intestine distal to the cecum—colon (Figure 1A). Intestinal samples for histology were taken from the middle of each intestinal segment. As previously described, samples were stained with H&E or Alcian Blue and Periodic Acid Schiff (AB-PAS) (Figure 1B) to quantify Paneth cells (15, 19, 43). A single, blinded investigator evaluated the tissue under an Eclipse Nikon microscope at 40X and manually quantified only stain positive cells located below the transit amplifying zone of the intestinal crypts. Results were cross-checked by the senior author for consistency. Paneth cells were quantified per crypt for at least 100 crypts per sample. While we have previously shown AB-PAS staining to be sufficient to quantify Paneth cells in rodents (12, 14, 19, 20), we have not done so in sheep. To confirm our AB-PAS staining was adequately capturing Paneth cells, we performed immunohistochemistry for lysozyme. Samples were treated with 10% goat serum (Zymed) for 30 min followed by exposing samples to anti-lysozyme (Dako) antibody at 4°C overnight. Anti-rabbit horseradish peroxidase (Dako) was applied to slides for 30 min, and samples were developed using a DAB substrate kit (Vector Labs) and counterstained with methyl green or Meyer’s hematoxylin Figure 2A).

*Statistical Analysis*: All experiments were performed at least triplicate, and specific sample sizes are denoted in the results section. ANOVA and non-parametric Kruskal–Wallis testing was performed to determine statistical significance using GraphPad Prism v9 (RRID: SCR_002798). Significance was set at *p* < 0.05 for all experiments.

## 3 Results

### 3.1 Paneth cell density increases proximally to distally along the length of the small intestine in newborn sheep

Representative histology images from intestinal samples harvested from term newborn sheep were stained with H&E for structure or with Alcian Blue-PAS staining for the presence of Paneth cells as previously described (Figure 1B) (15) and Paneth cell staining was confirmed by immunohistochemistry for anti-lysozyme (Figure 2A). Paneth cell density was significantly higher in the ileum than in the jejunum at term birth (*n* = 18 jejunal samples and *n* = 8 ileal samples, *p* < 0.0001). However, no Paneth cells were found in colonic samples (*n* = 18) (data not shown). To eliminate animal to animal variability, we quantified numbers from matched animals for whom we collected all three sections of the intestine and found similar distributions (Figure 2C).

### 3.2 Paneth cell density increases during gestation

Ileal samples from preterm lambs (126–127 days of gestation) and compared to samples from term lambs (140–141 days of gestation). Preterm lamb ileum had significantly fewer Paneth cells than term animals, as seen in Figure 2D (*p* = 0.013, *n* = 15 preterm and 8 term samples). Furthermore, linear regression analysis demonstrated a significant linear correlation between ileal Paneth cell density and gestational age (Figures 2E *p* = 0.016, R^2^ = 0.25, Sy.x = 0.7).

### 3.3 Paneth cell density in the immature ovine intestine decreases following bradycardic birth stress

**Figure 3:**
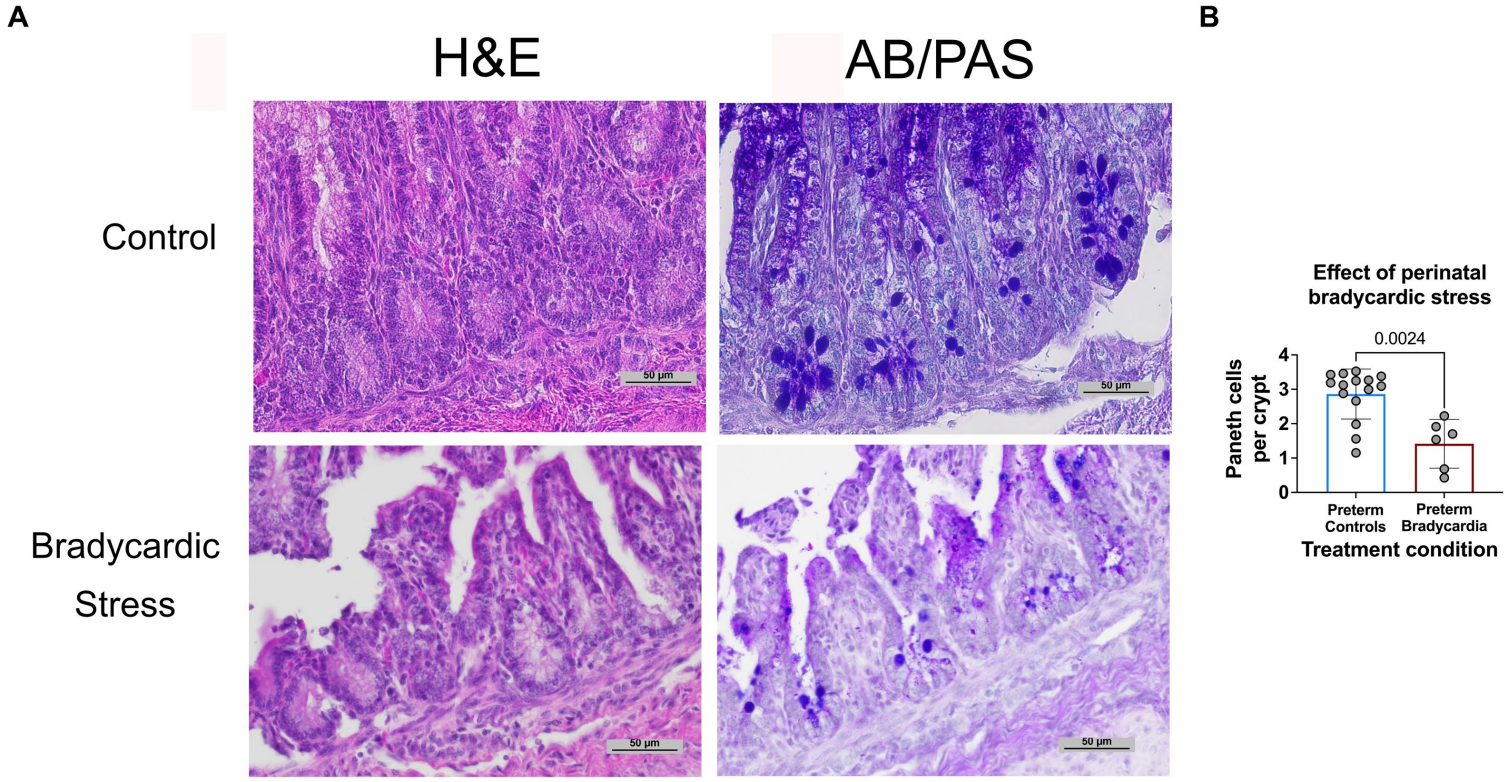
Prenatal bradycardic stress is associated with decreased Paneth cell density. Ileal Paneth cell density was quantified for preterm sheep who had experienced fetal bradycardia (127 days gestation, *n* = 6) and compared to the Paneth cell density of age-matched preterm controls (126–127 days gestation, *n* = 15, Figure 3A). **(A)** Representative histology from control and bradycardic stress groups using both H&E and AB/PAS staining. **(B)** Preterm sheep who had experienced perinatal bradycardia show statistically significant depletions of Paneth cell densities (1.62 Paneth cell/crypt compared to 3.13 Paneth cell/crypt, *p* = 0.002).

The relationship between birth asphyxia and stress and Paneth cell density has not been previously established in sheep. Therefore, to examine this relationship, intestinal samples were obtained at birth from preterm lambs who had experienced perinatal bradycardia. This preterm bradycardic group was compared to age-matched sham controls ventilated without bradycardia (Figure 3). Preterm lambs in the bradycardic (birth stress) group had significantly decreased Paneth cell density compared to controls (*p* = 0.0026, *n* = 6 preterm bradycardia, and *n* = 15 preterm controls from Figure 3).

## 4 Discussion

The immature intestine and the depletion of Paneth cell quantities have been linked to the development of NEC (3, 6). While rodents are often used to model Paneth cell distributions and densities to study intestinal diseases such as NEC, such models are not perfect. They can have several disadvantages, such as their size and intestinal development related to birth (21, 23, 24). Our study sought to examine the distribution and development of Paneth cells in an ovine model. Sheep have helped understand inflammatory diseases of the fetus and newborn (35, 36) and thus may be a feasible model to understand Paneth cell biology in the fetal and neonatal periods. Understanding Paneth cell biology in sheep models enables us to assess the impact of interventions such as feed type, mesenteric blood flow, and blood transfusions (44). We show that Paneth cell distribution patterns at birth in an ovine model closely resemble humans and mice, with the highest concentrations in the ileum, and are similarly gestation-dependent. Furthermore, our studies demonstrate that brief perinatal stress induced by bradycardia leads to significant depletion in Paneth cell density.

Prior work by Ergün et al. suggested that Paneth cell distribution throughout the sheep intestine was heterogeneous, with the greatest density occurring in the jejunum (38). However, this study was performed on 7 healthy adult sheep. In addition, Paneth cells were quantified by staining with phloxine-tartrazine and counting positive cells in ten crypts in five sections for each intestinal region. Flores et al. describe a morphological study in preterm lamb intestine, but the data on Paneth cells were limited to mRNA quantification on the limited number of lambs (39). Our study utilized Periodic acid Schiff and Alcian blue staining confirmed with immunohistochemistry against lysozyme to quantified Paneth cells over 100 crypts for each intestinal sample and in multiple sections across the preterm and term intestine. We confirmed our hypothesis that ovine Paneth cell distribution is similar to rodents and humans, with the highest concentration of Paneth cells in the ileum. These data contrast what was previously found by Ergün et al. However, it is reasonable that these differences may be due to the animal’s age as further proximal Paneth cell development may occur as the animal ages and its food source changes.

In humans and mice, Paneth cell development and quantity are directly correlated to increasing stages of development (8, 21, 22). However, this finding has not previously been studied in sheep. Our novel data show that Paneth cell concentrations increase with gestational age in a linear fashion, similar to what is seen in humans and rodents. For comparison purposes, preterm sheep between 123–126 days of gestation had an average of 3 Paneth cells per crypt which is similar to 14 day old C57Bl/6 mice born and human fetuses between 24 and 29 weeks (Stanford and Heida 2016). Simiarly, sheep at term equivalent had 4 Paneth cells per crypt which is similar to 24 day old mice and human fetuses 29–32 weeks of gestation. In other large animal models, such as the pig, the presence of Paneth cells continues to be debated; without consistent confirmation of their existence in the small intestine, the pig is not an ideal model for Paneth cell biology (3, 45, 46).

Lastly, we had the opportunity to examine preterm lambs with induced transient asphyxia leading to fetal bradycardia. Neonatal transient hypoxic-ischemia is a devastating and common cause of severe neurologic deficits in children impacting 1.5/1000 live births (47). While most information centers around the impact of transient hypoxia on the neurologic organs, there is limited data that this also impacts the intestine (48). Our lab has also previously shown that fetal exposures can impact Paneth cell density in offspring (49–51). Thus, we sought to determine if lambs exposed to transient asphyxia at or around the time of birth would impact Paneth cell numbers. Our data show that brief fetal stress caused by the induction of bradycardia correlated with lower Paneth cell numbers. While we do not yet have a mechanism behind this association, it is consistent with what we have found with fetal exposure to LPS-induced inflammation and requires further study. In addition, the association with ischemia and hypoxia/asphyxia with NEC is known, and Paneth cell biology may potentially have a role to play in this pathogenesis (1, 2).

There are several limitations to our study. The lamb ventilation protocols were part of different studies, and intestinal samples were collected over 10 years. However, one author (SFG) consistently dissected and collected all samples for consistency. We did not have a bradycardic perinatal stress protocol in term lambs, and our data are limited to preterm lambs.

Our novel findings support the possibility of utilizing ovine models for understanding Paneth cell biology in the fetus and neonate. Further, they contrast with previous descriptions of adult ovine Paneth cell development and show that the development and distribution of the Paneth cell throughout an ovine small intestine resemble what is seen in humans. In both species at birth, the highest concentrations of Paneth cells are in the ileum, and Paneth cell quantities were directly correlated to increasing gestational age.

Studies of Paneth cell function in humans and rodents have included measures of mRNA and protein expression of Paneth cell products (e.g., defensins, cathelicidins, lysozyme) and have shown maturation in parallel with development. The next steps in studying Paneth cell biology in the developing ovine intestinal tract would include identifying ovine Paneth cell products and developing antibodies to allow ovine Paneth cell function quantification.

## Data availability statement

The raw data supporting the conclusions of this article will be made available by the authors, without undue reservation.

## Ethics statement

The animal study was approved by The Institutional Animal Care and Use Committee (IACUC) at the State University of New York at Buffalo. The study was conducted in accordance with the local legislation and institutional requirements.

## Author contributions

GB: Formal analysis, Methodology, Project administration, Supervision, Validation, Visualization, Writing – review & editing. AC: Formal Analysis, Writing – review & editing. RS: Formal analysis, Methodology, Writing – original draft. SL: Methodology, Writing – review & editing, Conceptualization, Funding acquisition, Investigation. MP: Formal analysis, Methodology Writing – review & editing, Data curation. PC: Methodology, Supervision, Writing – review & editing, Formal analysis, Funding acquisition, Resources. SG: Conceptualization, Investigation, Methodology, Writing – review & editing, Supervision. MU: Conceptualization, Investigation, Methodology, Supervision, Writing – review & editing. SM: Conceptualization, Formal analysis, Funding acquisition, Investigation, Project administration, Resources, Software, Supervision, Validation, Writing – original draft, Writing – review & editing.
